# WeChat-based remote follow-up management reduces the burden of home care and anxiety on parents of children with refractory epilepsy: A randomized controlled study

**DOI:** 10.1097/MD.0000000000034070

**Published:** 2023-06-23

**Authors:** Xiaofen Huang, Yueming Kang, Meixin Wang, Qianqian Liu, Feng Wang, Mingzhu Zeng

**Affiliations:** a Department of Neurosurgery, the First Affiliated Hospital, Fujian Medical University, Fuzhou, Fujian, China; b Department of Neurosurgery, National Regional Medical Center, Binhai Campus of the First Affiliated Hospital, Fujian Medical University, Fuzhou, China.

**Keywords:** anxiety, burden of home care, epilepsy, remote follow-up management, WeChat

## Abstract

The present research was designed to study the effect of WeChat-based remote follow-up management on the burden of home care and anxiety on parents of children with refractory epilepsy.

**Methods:**

161 refractory epileptic children were included in this study. They were divided into control group and WeChat group according to their management protocols after discharge, namely, control group with traditional follow-up (n = 81) and WeChat group with remote follow-up based on WeChat (n = 81). We evaluated home care burden by family caregiver task Inventory (FCTI) scale and zarit burden interview (ZBI) scale, and evaluated negative emotion by self-rating anxiety Scale (SAS) scale and self-Rating depression scale (SDS) scale.

**Results:**

There was no significant difference in the demographic characteristics of epileptic children and their parents and the scores of FCTI, ZBI, SAS and SDS before treatment between the 2 groups (all *P* > .05), and the score of FCTI (20.86 ± 4.26), ZBI (39.63 ± 4.46), SAS (44.49 ± 4.15) and SDS (50.02 ± 4.13) in WeChat group were all significantly lower than the score of FCTI (25.25 ± 3.71), ZBI (45.47 ± 4.61), SAS (52.75 ± 4.93) and SDS (54.51 ± 6.59) in control group (all *P* < .05).

**Conclusion:**

WeChat-based remote follow-up management reduces the burden of home care and anxiety on parents of children with refractory epilepsy.

## 1. Introduction

Epilepsy, one of the most common neurological diseases, is a chronic disease that results in transient brain dysfunction due to sudden abnormal discharge of brain neurons.^[1]^ There are about 70 million epilepsy patients in the world and 9 million in China, about 90% of epilepsy patients were in developing countries.^[2,3]^ Epilepsy has become the second most common disease in neurology after headache. Statistical data show that about 30% of epileptic patients are still difficult to control after planned antiepileptic drug treatment, calling intractable epilepsy or refractory epilepsy.^[4,5]^ Surgery is the main treatment option for patients with intractable epilepsy, but patients need to continue to take antiepileptic drugs for a long time after surgery to achieve ideal disease control effect.^[6,7]^ However, postoperative antiepileptic drug treatment and rehabilitation of children with epilepsy is a long and arduous process, and the parents of the child is under great psychological pressure and prone to various psychological problems in this process, among which anxiety is the most common.^[8,9]^ Importantly, the psychological problems of the family can directly affect the recovery of the child, so professional medical services are meaningful for these parents.^[10,11]^

Continuity of care is designed through a series of actions to ensure that patients receive different levels of collaborative and continuous care in different health care settings (e.g., from hospital to home) and within the same health care setting (e.g., different departments of a hospital), usually referring to the continuation from hospital to home, including discharge planning developed by the hospital, referral, continuous follow-up and guidance after the patient return to the family or community.^[12,13]^ As a part of continuous nursing care, social software is widely proved to be beneficial to patients after discharge, such as breast cancer,^[14,15]^ non-small cell lung cancer,^[16]^ orthopedic patients^[17]^ and premature infants.^[18]^ WeChat, with 1.2 billion users, is the most commonly used social APP for Chinese people. WeChat follow-up nursing methods are widely used by nursing staff in China for medication and rehabilitation monitoring, psychological status assessment, and health knowledge education after discharge.^[19,20]^ To our knowledge, none of the studies reported on WeChat-based models of postoperative care for children with epilepsy. Therefore, we designed to study the effect of WeChat-based remote follow-up management on the burden of home care and anxiety on parents of children with refractory epilepsy in this study.

## 2. Methods

### 2.1. Ethical statement and sample size calculation

The present study was approved by the ethics Committee of the First Affiliated Hospital of Fujian Medical University (Approval No.2019274), and we also abided by the principles of the Helsinki Declaration. In addition, all parents of recruited epileptic children were informed of the detailed agreement of this study and their rights and obligations, and all of them signed informed consent form. Based on the data from our previous study^[18]^ and assuming that the alpha value was set at 0.05 with a power of 0.9, the required number of participants was calculated to be 68 in each group. Assuming a 10% missing rate, the total sample size was set as at least 150, each with 75 cases at least in the intervention group and the control group.

### 2.2. Research design and randomization

This study was a single center randomized controlled study that conducted in department of Neurosurgery, the First Affiliated Hospital of Fujian Medical University. We numbered the patients of recruited epileptic children according to the order in which they were recruited for the study and grouped them using a random number table method. The recruited epileptic children were divided into control group and WeChat group according to 1:1 agreement after. WeChat-based remote follow-up management was performed in the WeChat group after discharge, while traditional outpatient follow-up management was performed in the control group. On the day of discharge and 6 months after discharge, we evaluated home care burden by family caregiver task inventory (FCTI) scale and zarit burden interview (ZBI) scale, and evaluated negative emotion by self-rating anxiety Scale (SAS) scale and self-rating depression scale (SDS) scale on parents of epileptic children.

Inclusion criteria: Epileptic seizures more than 10 times/year; 2 to 10 years old; Epilepsy duration is more than 12 months; Normal intelligence; Living with father and mother, and younger than 40 years old. Exclusion criteria: Accompanied by other congenital diseases or nervous system diseases; Postoperative complications, such as infection, hydrocephalus, etc. Contralateral hemisphere dysfunction; Mental retardation; Parents of epileptic children will not use WeChat.

### 2.3. Intervention methods

Control group Father or mother of epileptic children in the control group received routine outpatient follow-up management for 6 months. In detail, the responsible nurse shall register the medical records before discharge, explain the purpose, role and importance of continuous medication after discharge to the parents of the children, follow up the parents of the children every 2 weeks by telephone, and require outpatient follow-up every month.

WeChat group Father or mother of epileptic children in the control group received WeChat-based remote follow-up management for 6 months. Detailly: The responsible nurse shall register the medical records before discharge, and require outpatient follow-up every month. Set up a special WeChat account to add children parents as WeChat friends, and establish a WeChat public platform for epilepsy patients. Question and answers module: The medical staff of the team replied online to the parents of epileptic children from 18:00 to 21:00 every day, and reminded them one by one of their medication and outpatient follow-up. Epilepsy knowledge education module: Publish at least 1 piece of epilepsy treatment/ nursing related knowledge and health education content on WeChat platform every day in the form of video, pictures, etc. Medication reminder module: Every day, 1 team member issues an electronic medication tracking record on the WeChat platform and urges patients in the WeChat group to fill in the medication record in a timely manner.

### 2.4. Research tool

We used FCTI scale and ZBI scale to assess burden of home care in father or of mother epileptic children, and used SAS scale and SDS scale to assess negative emotion in in father or of mother epileptic children at time of discharge and 6 months after discharge. The details contained in the FCTI, ZBI, SAS and SDS scales have been described in previous study by our team.^[18]^

### 2.5. Statistical analysis

Excel tables are used to collate the data of epileptic children and their parents, and SPSS25.0 (IBM) is used to statistically analyze these data. The measurement data is expressed as (mean ± standard deviation), and the Student *t* test is used to compare the differences between the 2 groups of measurement data. The counting data showed no percentage, and chi square test was used to compare the difference between the 2 groups of counting data. *P* < .05 indicates that the difference is statistically significant.

## 3. Results

### 3.1. Demographic characteristics of subjects

The demographic characteristics of epilepsy children and their fathers or mother were showed in Table 1. In detail, there was no significantly difference between control group and WeChat group on epileptic children, such as gender, age, epilepsy onset age, only child family, epileptic drugs and medical payment method. At the same time, there was no significantly difference between control group and WeChat group on parents of children with epilepsy, such as age, gender, education level, residential address and concern about side effects of epilepsy drugs.

**Table 1:** Demographic characteristics of epilepsy children and their fathers or mother.

Group	WeChat group (n = 81)	Control group (n = 81)	t/χ^2^ test	*P* value
Epilepsy children				
Male (n (%))	51 (62.96)	48 (59.26)	0.234	.629
Age (yr)	4.85 ± 1.21	4.61 ± 1.14	1.269	.206
Epilepsy onset age (yr)	3.12 ± 0.78	2.97 ± 0.71	1.335	.184
Only child family (n (%))				
Yes	51 (62.96)	46 (56.79)	0.642	.423
No	30 (37.04)	35 (43.21)		
Epileptic drugs (n (%))				
Yes	51 (62.96)	53 (65.43)	0.107	.749
No	30 (37.04)	28 (34.57)		
Medical payment method (n (%))				
Partly own expense	11 (13.58)	10 (12.35)	0.055	.815
Insurance	70 (86.42)	71 (87.65)		
Father or of mother epileptic children				
Male (n (%))	14 (17.28)	11 (13.58)	0.426	.514
Age (yr)	33.33 ± 4.13	33.18 ± 4.11	0.231	.818
Education level (n (%))				
Under high school	28 (34.57)	27 (33.33)	0.955	.812
High school	15 (18.52)	20 (24.69)		
Junior college	20 (24.69)	18 (22.22)		
Junior college	18 (22.22)	16 (19.75)		
Residential address				
Rural area	30 (37.04)	33 (40.74)	0.234	.629
City	51 (62.96)	48 (59.26)		
Concern about side effects of epilepsy drugs (n (%))				
Yes	61 (75.31)	57 (70.37)	0.499	.480
No	20 (24.69)	24 (29.63)		

### 3.2. Burden of home care in father or of mother epileptic children

After epileptic children were included in this study, we evaluated their father or mother home care burden by FCTI scale and ZBI scale, and found that the score of FCTI scale (31.79 ± 3.33) and ZBI scale (51.97 ± 3.90) in WeChat group had no significantly difference with the score of FCTI scale (31.10 ± 4.21) and ZBI scale (25.25 ± 3.71) in control group (all *P* > .05) (Table 2).

**Table 2:** Comparison of FCTI and ZBI scale scores of parents of 2 groups of epileptic children before and after intervention.

Group		WeChat group (n = 81)	Control group (n = 81)	*t* test	*P* value
Score of FCTI	Pre-intervene	31.79 ± 3.33	31.10 ± 4.21	1.159	.248
	Post-intervene	20.86 ± 4.26*	25.25 ± 3.71*	6.980	<.001
Score of ZBI	Pre-intervene	51.97 ± 3.90	51.00 ± 4.34	1.505	.134
	Post-intervene	39.63 ± 4.46*	45.47 ± 4.61*	8.189	<.001

Note: Compared with pre- intervene.

FCTI = family caregiver task inventory, ZBI = zarit burden interview.

* *P* < .05.

Six months after intervention of different schemes, we evaluated again their father or mother home care burden by FCTI scale and ZBI scale. And we found that compared with before intervention, FCTI scores and ZBI scores of parents of epileptic children in 2 group decreased, and the score of FCTI scale (20.86 ± 4.26) and ZBI scale (39.63 ± 4.46) in WeChat group were all significantly lower than the score of FCTI scale (25.25 ± 3.71) and ZBI scale (45.47 ± 4.61) in WeChat group (all *P* < .05) (Table 2).

### 3.3. Negative emotion scores for father or of mother epileptic children

After epileptic children were included in this study, we evaluated their father or mother negative emotion by SAS scale and SDS scale, and found that the score of SAS scale (59.26 ± 4.73) and SDS scale (58.07 ± 4.83) in WeChat group had no significantly difference with the score of SAS scale (59.51 ± 5.04) and SDS scale (57.43 ± 4.38) in control group (all *P* > .05) (Table 3).

**Table 3:** Comparison of negative emotion scores between 2 groups of epileptic children parents before and after intervention.

Group		WeChat group (n = 81)	Control group (n = 81)	*t* test	*P* value
Score of SAS	Pre-intervene	59.26 ± 4.73	59.51 ± 5.04	0.321	.748
	Post-intervene	44.49 ± 4.15*	52.75 ± 4.93*	11.533	<.001
Score of SDS	Pre-intervene	58.07 ± 4.83	57.43 ± 4.38	0.866	.377
	Post-intervene	50.02 ± 4.13*	54.51 ± 6.59*	5.202	<.001

Note: Compared with pre- intervene.

SAS = self-rating anxiety scale, SDS = self-rating depression scale.

* *P* < .05.

Six months after intervention of different schemes, we evaluated again their father or mother home care burden by SAS scale and SDS scale. And we found that compared with before intervention, SAS scores and SDS scores of parents of epileptic children in 2 group decreased, and the score of SAS scale (44.49 ± 4.15) and SDS scale (50.02 ± 4.13) in WeChat group were all significantly lower than the score of SAS scale (52.75 ± 4.93) and SDS scale (54.51 ± 6.59) in WeChat group (all *P* < .05) (Table 3).

## 4. Discussion

In China, there are 9 million epilepsy patients and 400,000 new epilepsy patients every year, and the incidence of epilepsy in Chinese population is 0.5% to 0.75%, of which about 30% are refractory epilepsy, and more than 80% of epilepsy onset age is preschool.^[21,22]^ Refractory epilepsy has a great influence on the growth and development of children, and its pathogenesis may be related to factors such as focal cortical dysplasia, glial cell proliferation, neuronal loss, and lichen fiber budding.^[23,24]^ Surgery is a very important treatment for children with drug-refractory epilepsy, and many domestic studies have confirmed that surgical treatment have a good effect on children with refractory epilepsy.^[6,7]^ It is important to note that children with epilepsy still need to take antiepileptic drugs for a long time after surgery to control seizures. Unreasonable postoperative care of children with epilepsy may cause an increase in the number of seizures, affect the growth and development of the child, threaten the life of the child in severe cases, and seriously reduce the quality of life of the child family. Parents, as the primary caregivers of children with epilepsy, often feel stressed and anxious after the child surgical treatment and discharge from the hospital due to lack of epilepsy care experience and medical support from professional medical staff.^[25,26]^ Therefore, how to improve the care ability of parents and increase access to medical support for children with epilepsy to enable children with epilepsy to obtain better home care is a concern of medical professionals.

In this study, we found that the FCTI scores (31.79 ± 3.33 and 31.10 ± 4.21) and ZBI scores (51.97 ± 3.90 and 51.00 ± 4.34) at discharge of the 2 groups indicated that the parents of children with epilepsy faced a heavy burden of family care.^[18]^ Moreover, results of the present study also showed that the SAS scores and SDS scores of the parents of the children with epilepsy were (59.26 ± 4.73 and 59.51 ± 5.04) and (58.07 ± 4.83 and 57.43 ± 4.38), respectively. According to the interpretation of the SAS scale and the SDS scale, that is, SAS score >50 indicates anxiety^[27]^ and SDS score higher than 40 indicates depression,^[28]^ and parents of children with epilepsy in both groups have different degrees of anxiety and depression at the time of discharge.

Burden of home care refers to the negative experience of unpleasantness and discomfort caused by the stress suffered by caregivers in the process of caring for patients, including the physical, psychological, emotional, economic and social life of caregivers. The impact of children with epilepsy on home caregivers is first manifested in the long care time, the limited hospitalization time of epilepsy patients, and the parents bear the main nursing care work for most of the time after discharge, which has a great burden on the physical, psychological and time aspects of the parents of the children.^[29,30]^ On the other hand, the seizures of epilepsy do not have any regularity, most of the seizures have consciousness disorders, forget the entire seizure process, coupled with the young age of the child, lack of judgment of danger, the risk of accidental injury is high, so daily life needs special care for people.^[29,30]^ In addition, children with epilepsy need to take anti-epileptic drugs regularly for a long time after surgery, but the children lack understanding of the disease, low medication compliance, and if they are not supervised, they may miss or take it by mistake. All in all, Children with epilepsy, especially a child with epilepsy treated by surgery, is a serious negative event for a family, and home caregivers not only need to take care of the child in all aspects, around the clock, to deal with emergencies anytime, anywhere, but also to worry about the prognosis of the disease and a variety of adverse reactions of anti-epileptic drugs.^[31,32]^

Fortunately, we found that continuity of care after discharge, whether routine in control group or WeChat-based remote management in WeChat group, all reduced FCTI, ZBI, SAS, and SDS scores for home caregivers, suggesting continuous nursing is beneficial to reduce the care burden and negative emotions of family nurses for children with epilepsy after discharge. Importantly, we also found that the score of FCTI, ZBI, SAS and SDS in WeChat group were all significantly lower than the score of FCTI, ZBI, SAS and SDS in control group at the time of 6 months after discharge, indicating that the WeChat based remote follow-up management is more beneficial than routine care to reduce the home care burden and negative emotions of family nurses for children. Previous studies have found that as a telemedicine tool, WeChat-based remote follow-up management is beneficial for disease control and recovery of chronic disease patients and cancer patients, and can reduce medical costs, reduce the burden of family caregivers and improve the quality of life of patients’ families (patients and their home caregivers).^[33,34]^

Some of the limitations of our study should be noted. First, as a single center study, this study included a low sample size and had regional restrictions. Second, the remote follow-up management based on WeChat is limited for some parents of epileptic children, so many parents are not included, which leads to artificial bias. At last, the long-term impact of WeChat based remote follow up management on epileptic children, such as seizure frequency, medication compliance and quality of life, should be studied in the future.

## 5. Conclusion

WeChat-based remote follow-up management reduces the burden of home care and anxiety on parents of children with refractory epilepsy.

## Acknowledgments

Thank all parents of epileptic children who participated in this study, doctors and nurses in our hospital for their support to this study; Thank Dr Yan Zheng of Fuzhou First Hospital for her guidance.

## Author contributions

**Conceptualization:** Meixin Wang.

**Data curation:** Xiaofen Huang, Yueming Kang, Meixin Wang, Qianqian Liu, Feng Wang, Mingzu Zeng.

**Formal analysis:** Xiaofen Huang, Feng Wang.

**Funding acquisition:** Mingzu Zeng.

**Methodology:** Xiaofen Huang.

**Software:** Qianqian Liu.

**Writing – original draft:** Feng Wang.

**Writing – review & editing:** Mingzu Zeng.
